# Anion‐Selective Layered Double Hydroxide Composites‐Based Osmotic Energy Conversion for Real‐Time Nutrient Solution Detection

**DOI:** 10.1002/advs.202103696

**Published:** 2022-01-06

**Authors:** Yaqian Liu, Jianfeng Ping, Yibin Ying

**Affiliations:** ^1^ Laboratory of Agricultural Information Intelligent Sensing School of Biosystems Engineering and Food Science Zhejiang University Hangzhou Zhejiang 310058 China

**Keywords:** anion‐selective, layered double hydroxide, nanofluidic channels, nutrient solution, osmotic energy

## Abstract

Nanofluidic channels based on 2D nanomaterials are promising to harvest osmotic energy for their high ion selectivity and osmotic conductivity. However, anion‐selective nanofluidic channels are rare and chemical modification is necessary through fabrication. Here, a naturally anion‐selective composite membrane is reported, that is, NiAl‐Layered double hydroxide (LDH) coated anodic aluminum oxide (LDH@AAO), using a simple precipitant‐free in situ growth technique. Positively charged LDH plates growing in channels of AAO function as screening layers for anions. Both experiments and theoretical simulations are enforced to certify the vital role of LDH growth in ion distribution and salinity gradient energy conversion. The composite membrane achieves high output performance and long‐term stability. Furthermore, novel applications of nanofluidic channels are explored in hydroponic production and design a real‐time detecting system based on LDH@AAO composite membranes for nutrient solution. This work provides insights into naturally anion‐selective nanofluidic channels for osmotic energy harvesting and broadens the application in agricultural information sensing.

## Graphical abstract

An anion‐selective nanofluidic membrane, that is, layered double hydroxide (LDH) coated anodic aluminum oxide (LDH@AAO), is reported for osmotic energy conversion. The membrane exhibits excellent output performance. Experiments and theoretical simulations are enforced to certify the vital role of LDHs. A real‐time detecting system for nutrient solution management in hydroponic production is established based on LDH@AAO.

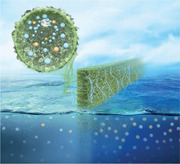

## 1 Introduction

As a type of blue energy, salinity gradient energy exists between two solutions with different salinities, which is common in seawater and river water. Considering an increasing number of miniaturized sensors in daily life demand sustainable and environmental‐friendly power, exploiting salinity gradient energy is of great value. Pressure‐retarded osmosis and reversed electrodialysis are two main traditional approaches to harvesting salinity gradient energy. However, the development of these two technologies is limited due to undesirable power density, high energy costs, and intrinsic trade‐off between power output and energy harvesting efficiency.^[^ ^1^ ^]^ This limitation mainly results from the lack of appropriate membranes able to allow for a sufficient flow.^[^ ^2^ ^]^ Inspired by biological ion channels embedded in lipid bilayers in living organisms, the occurrence of nanofluidic channels has large potential in realizing transmembrane ion flow efficiently.^[^ ^3^ ^]^

Existing nanofluidic channels applied to osmotic energy harvesting consist of single‐pore‐ and membrane‐based channels. Although single‐pore‐based nanofluidic channels exhibit ultrahigh output power density (10^3^–10^6^ W m^−2^), it is challenging to translate the high power density into real high power with porous membranes.^[^ ^4^ ^]^ To gain the promoted output performance, high surface charge density, large surface area, and nanoscale channel sizes are necessary for membrane‐based nanofluidic channels.^[^ ^5^ ^]^ Many two dimensional (2D) materials including graphene, hexagonal boron nitride, MXene, graphene oxide, and molybdenum disulfide have been exploited in membrane‐based nanofluidic channels for osmotic energy harvesting recently in view of their superiority in electrical and mechanical properties.^[^ ^6^, ^7^, ^8^, ^9^, ^10^, ^11^, ^12^, ^13^, ^14^, ^15^ ^]^ However, almost all of them are naturally cation‐selective membranes due to hydroxide adsorption of most 2D materials. Considering anion‐selective membranes and cation‐selective membranes are of the same value as necessary components for reverse electrodialysis (RED), advances in anion‐selective membranes are still required for large‐scale application of RED in future. To obtain anion‐selective membranes, surface modification with chemicals is a common choice, which adds complexity during the fabrication process.^[^ ^12^, ^16^, ^17^ ^]^ Layered double hydroxides (LDHs) have attracted widespread attention in optical sensors, water splitting, supercapacitors, electrocatalytic oxygen evolution, lithium batteries, and biomedical applications recently.^[^ ^18^, ^19^, ^20^, ^21^, ^22^ ^]^ Positively charged layers of LDHs equip them with the capability to act as functional layers in anion‐selective nanofluidic channels.

**Figure 1:**
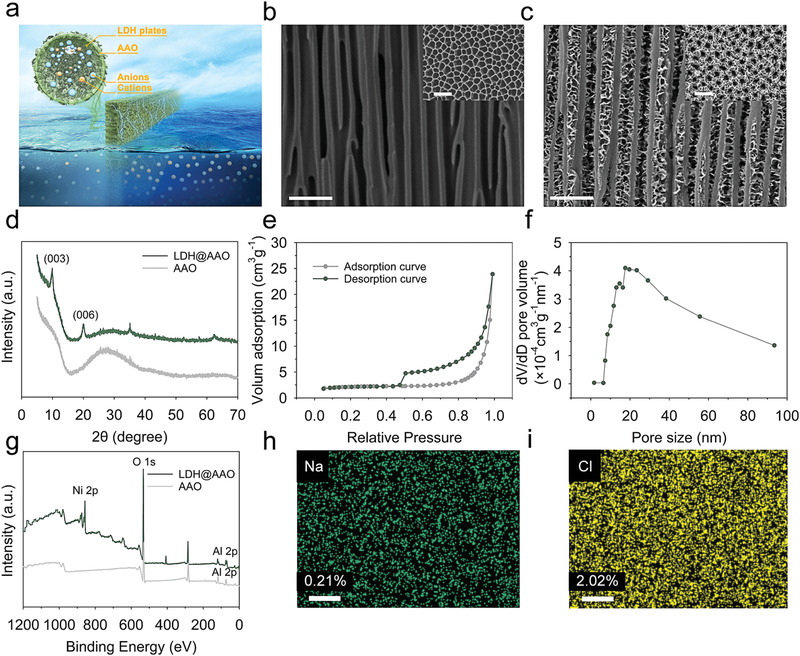
LDH@AAO composite membranes. a) Schematic showing osmotic power generation with the LDH@AAO composite membrane. LDH plates are distributed on the outer and inner surfaces of the AAO channels. The salinity gradient drives both anions and cations flow from a higher to a lower concentration. However, the positive surface charge brought by LDH contributes to a higher population of anions (blue balls) in nanofluidic channels and repels the entrance of cations (orange balls). The net movement of anions in the composite membrane results in the observation of a current. b,c) Cross‐sectional SEM images of the AAO template and the LDH@AAO composite membrane (scale bar: 500 nm). Inset displays the outer surface (scale bar: 500 nm). d) XRD patterns of the AAO template and the LDH@AAO composite membrane. e) Nitrogen adsorption/desorption isotherms and f) corresponding pore size distribution of the LDH@AAO composite membrane. g) XPS spectrum of the AAO template and the LDH@AAO composite membrane. h,i) EDX mapping of Na and Cl on the cross‐section of the LDH@AAO composite membrane (scale bar: 1 µm).

Here, we report an anion‐selective composite membrane, that is, NiAl‐LDH coated anodic aluminum oxide (LDH@AAO), for harvesting salinity gradient energy (**Figure** **1**a). On one hand, NiAl‐LDH growing on surfaces of AAO provides sufficient positive charge density to screen cations. The distribution of NiAl‐LDH in the inner surfaces of AAO also tunes the nanochannel size to an appropriate range so that an excellent ion selectivity could be achieved. On the other hand, AAO participates in the formation of NiAl‐LDH in Ni(NO_3_)_2_ 6H_2_O aqueous solutions. It is also the backbone of the LDH@AAO composites. When mounted between artificial seawater (0.5 m NaCl) and artificial river water (0.01 m NaCl), the fabricated membrane can harvest salinity gradient energy up to 2.85 W m^−2^.

Importantly, we first developed the application of nanofluidic channels in real‐time detecting ion concentration of nutrient solutions in hydroponic production. Hydroponic production has been a popular choice in agriculture.^[^ ^23^ ^]^ Crops are grown in nutrient solutions without soil in this way. Nutrient solutions typically consist of multiple fertilizer ions and provide crops with the necessary nutrients. It is vital to monitor the ion concentration in nutrient solutions because the yield will be limited when the concentration is too low or too high.^[^ ^24^ ^]^ Electrical conductivity (EC) is commonly used to reflect the total amount of ions in nutrient solutions. However, long‐term immersion in nutrient solution tends to pollute the electrode for conductivity measurement, which burdens users with the demand for regular calibration and replacement of electrodes. Also, high costs arise from high density of sensors per unit of area for real‐time monitoring.^[^ ^25^ ^]^ The capability of nanofluidic channels in converting salinity gradient energy into electricity makes it possible to employ electrical signals generated from nanofluidic channels as a measurement of ion concentration levels in nutrient solutions without an external power supply. We demonstrated a real‐time detecting system based on LDH@AAO composite membranes for the nutrient solution. The measured current is positively correlated to the ion concentration of nutrient solutions. When the measured current is beyond the appropriate range for the growth of crops, the system will warn users to adjust the ion concentration in time.

## 2 Results

### 2.1 Characterization of LDH@AAO Composite Membranes

The LDH@AAO composite membranes are fabricated in the method of simple and precipitant‐free in situ growth.^[^ ^26^ ^]^ In order to gain the appropriate channel size of AAO, we employed AAO templates with different channel sizes and fabricated composite membranes (Figure S1, Supporting Information). When the channel size of AAO is smaller than 160 nm, the diffusion of Ni^2+^ into the AAO pores is hindered, which is the reason for the failure of nucleation of NiAl‐LDH and the formation of boehmite in channels. The channel size of AAO was then controlled in the range of 160 to 200 nm. Figure S2, Supporting Information, gives the photograph of the LDH@AAO composites, which is 1.2 cm in diameter and appears light green. Scanning electron microscopy (SEM) reveals the morphology and structure of the AAO template and NiAl‐LDH@AAO composite membranes (Figure 1b,c). The outer and inner surfaces of the AAO template are both distributed with LDH plates. Interlaced LDH plates shield pores on AAO templates. Large and dense LDH plates are parallel to each other along the channels in AAO. The pH of the reaction solution and reaction time exert great influence on the nucleation and growth of LDH (Figures S3 and S4, Supporting Information). The thickness of AAO is 43.3 µm while the thickness of the composite membrane is 45.8 µm (Figure S5a,b, Supporting Information). The distribution of LDH layers on the top and bottom surface of AAO is nearly symmetrical (Figure S5c,d, Supporting Information). The structure of AAO templates and LDH@AAO composite membranes were certified through X‐ray photoelectron spectroscopy (XPS) and X‐ray diffraction (XRD) (Figure 1d,g and Figure S6, Supporting Information). As for O 1s of AAO templates, two peaks at the binding energy (BE) of 531.0 and 532.0 eV corresponded to the characteristic of O^2−^ and OH^−^ ions respectively, which is consistent with the existence of lattice oxygen and hydroxyl ions in the composition of AAO templates. In contrast, the peak at BE of 532.1 eV is attributed to the surface OH^−^ ions of LDHs. The results are in agreement with previous research.^[^ ^27^, ^28^ ^]^ The diffraction peaks of LDH@AAO near 10° and 20° shown in the XRD pattern are assigned to the (003) and (006) planes of LDHs (Figure 1d). The nitrogen adsorption/desorption isotherms of the LDH@AAO composites in Figure 1e which is a typical type IV curve indicate that the composite membrane has a large uniform mesoporous structure. As shown in Figure 1f, the mean size of nanochannels in composite membranes is calculated to be 17.8 nm using the Barrett–Joyner–Halenda (BJH) model. After immersing the NiAl‐LDH@AAO composite membrane in 0.5 m NaCl for 24 h and removing unbound ions with deionized water, we conducted energy‐dispersive X‐ray spectroscopy (EDX) mapping on the cross‐section of the composite membrane (Figure 1h,i). The result demonstrates the selectivity of anions induced by positive surface charges of LDH plates. The composite membrane also exhibits great hydrophilicity as the contact angle is almost 41° (Figure S7, Supporting Information).

### 2.2 Evaluation of Surface‐Charge‐Governed Ion Transport

**Figure 2:**
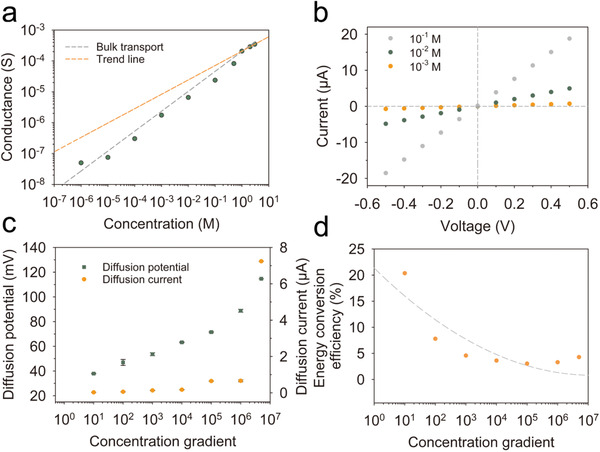
The ionic transport through the LDH@AAO composite membrane. a) The measured transmembrane ionic conductance as a function of NaCl concentration. Error bars represent SD (*n* = 3). b) Current‐voltage curves of LDH@AAO composite membrane for varying NaCl concentrations of 1–100 mm. c,d) Measured *E*_diff_, *I*_diff_ and calculated energy conversion efficiency under different salinity gradients. Error bars represent SD (*n* = 3).

We investigated the ion's transport properties of the LDH@AAO composites with an electrochemical cell (Figure S8, Supporting Information). The ion conductivity of the composite membrane was measured as a function of NaCl concentration (**Figure** **2**a). When the concentration is higher than 1 m, the conductivity of the composite membrane is mainly dependent on NaCl concentration, which is similar to the conductivity of bulk NaCl solution (dashed gray line). The further decrease of NaCl concentration increases the thickness of electric double layer, which is close to the width of the nanochannel.^[^ ^29^, ^30^ ^]^ As a result, the deviation of the ionic conductivity from the bulk value at low concentrations certifies the surface charge‐governed ion transport property of the LDH@AAO composites.^[^ ^7^, ^31^ ^]^ Figure 2b presents current‐voltage (*I*‐*V*) curves for a series of NaCl concentrations. The symmetric structure of the composite membrane contributes to the linear ohmic behavior without ionic current rectification. We further explored the ionic transport behaviors under various concentration gradients. One side of NaCl solution was fixed at 10^−6^ m, while the other side ranged from 10^−5^ to 5 m. A pair of salt bridges was used to eliminate the imbalanced electrode potential.^[^ ^12^, ^40^ ^]^ The intercepts on the current and voltage axes of *I‐V* curves are corresponding to the diffusion current (*I*_diff_) and potential (*E*_diff_) respectively. When the concentration gradient increases, the *E*_diff_ and *I*_diff_ both increase. The calculated energy conversion efficiency (*η*) sharply decreases from 20.4% to 3.3% as the concentration gradient increases from 10 to 5 × 10^6^ (Figure 2d, Note S1, Supporting Information).

### 2.3 Osmotic Energy Conversion of the Composite Membrane

**Figure 3:**
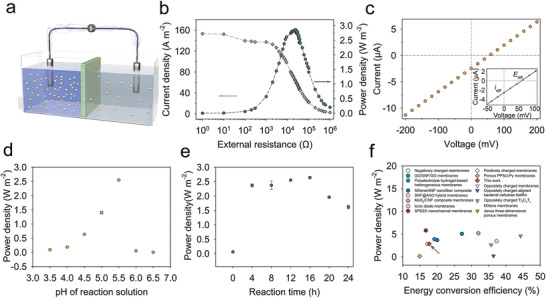
Osmotic energy conversion of the anion‐selective composite membrane. a) Schematic of the energy harvester. b) The current density and output power density under 0.5/0.01 m NaCl salinity gradient as the function of load resistance. The peak power density is 2.85 W m^−2^ when the load resistance is 23 kΩ. c) *I*‐*V* curves under a 50‐fold salinity gradient across the composite membrane. The inset of (c) displays the macroscopic view of the intercepts on the current and voltage axes, which are corresponding to *I*_diff_ and *E*_diff_ respectively. d) The power density of the composite membrane corresponding to different growth pH values with the reaction time fixed as 12 h(*R*_L_ = 30 kΩ). Error bars represent SD (*n* = 3). e) The power density of the composite membrane corresponding to different reaction times with growth pH fixed as 5.5 (*R*_L_ = 30 kΩ). Error bars represent SD (*n* = 3). f) Comparison of the output performance and energy conversion efficiency with reported nanofluidic membranes in the literature based on the same testing area. These membranes are classified into three categories including negatively charged membranes, positively charged membranes, and oppositely charged membranes.^[^ ^6^, ^7^, ^12^, ^16^, ^29^, ^31^, ^32^, ^33^, ^34^, ^35^, ^36^, ^37^ ^]^

To investigate the osmotic energy conversion capability of the LDH@AAO composites, we employed artificial seawater and river water to provide the salinity gradient. As shown in **Figure** **3**a, we connected an external load resistance (*R*_L_) to output the harvested energy. Figure 3b recorded the measured current which decreased when the external resistance increased. The electric power density, calculated as *P*_R_ = *I* ^2^ × *R*_L_, reached a maximum of 2.85 W m^−2^ when the load resistance was about 23 kΩ. The *I*‐*V* curve for a 50‐fold salinity gradient was illustrated in Figure 3c. The intercepts on the current and voltage axes are corresponding to *I*_diff_ and *E*_diff_ which are 2.46 µA and 55.7 mV respectively (the imbalanced electrode potential has been eliminated through the use of a pair of salt bridge). The corresponding energy conversion efficiency of the composite membrane calculated from these parameters is 17.6%. As stated before, the pH of the reaction solution and reaction time during the fabrication of composite membranes exert great influence on the nucleation and growth of LDH. These two parameters further affect the osmotic energy conversion behavior of the composite membrane since LDH plates mainly function as screening layers in nanochannels. To investigate the influence of pH, we fixed the reaction time at 12 h and recorded the corresponding power density for different pH in Figure 3d. The power density increases from 0.09 to 2.55 W m^−2^ when the pH increases from 3.5 to 5.5. The relatively high pH of the reaction solution is favorable for LDH growth in nanochannels, contributing to the increased surface charge density and strengthened anion selectivity. The continuous increase of pH leads to dense distribution of NiAl‐LDH on the surface of AAO, thus hindering the diffusion of Ni^2+^ into nanochannels and preventing the growth of LDH plates along nanochannels. The large steric hindrance to the transmembrane ion transport and relatively low charge density decreases the power density. This mechanism is also adaptable for the effect of the reaction time. We fixed the pH at 5.5 and obtained a peak value of power density as 2.64 W m^−2^ when the reaction time was 16 h (Figure 3e). Figure 3f compares the osmotic energy conversion capability of our LDH@AAO composite membrane with existing nanofluidic membranes. These membranes are classified into three categories according to their ion selectivity. The development of cation‐selective nanofluidic membranes is better than anion‐selective membranes. Advances in anion‐selective membranes are required for RED. As a novel anion‐selective nanofluidic membrane, the LDH@AAO membrane possesses relatively high energy conversion efficiency and output power density. The simple precipitant‐free in situ growth technique without chemical modification also makes the LDH@AAO membrane more appropriate for large‐scale application in the future.

### 2.4 The Effect of LDH Growth on the Ion Concentration Distribution and Energy Conversion

**Figure 4:**
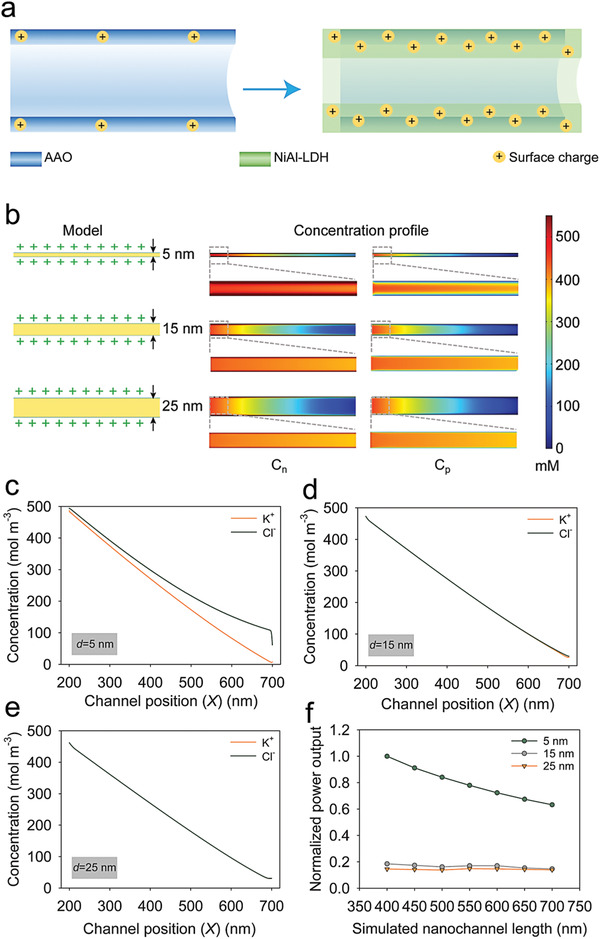
Effect of LDH growth in nanofluidic channels. a) LDH plates narrow the channel size, improve the surface charge density, and lengthen the channel length. b) The ionic concentration profiles of nanofluidic channels (*l* = 500 nm, *σ* = 0.08 C m^−2^) with various channel sizes based on numerical simulations. c–e) Average cation and anion concentration along the nanofluidic channel (*l* = 500 nm, *σ* = 0.08 C m^−2^) with various pore sizes including 5 (c), 15 (d), and 25 nm (e). The x‐coordinate is corresponding to the position of nanofluidic channels in the simulation. The *X* = 200 nm refers to the end of left electrolyte reservoir and the start of nanofluidic channel while the *X* = 700 nm refers to the end of nanofluidic channel and the start of right electrolyte reservoir. f) The calculated normalized power output of nanofluidic channels (*σ* = 0.08 C m^−2^) with various channel sizes and lengths.

In order to give further insight into the mechanism of power generation under the growth of LDH on AAO, numerical simulations were carried out based on Poisson and Nernst Planck (PNP) equations (Note S2, Supporting Information). Considering the growth of LDH in nanofluidic channels narrows the channel size (*d*), improves the surface charge density (*σ*), and extends the channel length (*l*) (**Figure** **4**a), we then focused on the influence of these three parameters. The nanofluidic channel was simplified into a 2D model as shown in Figure S9, Supporting Information, to simulate the ion transport. Figure 4b displays the concentration distribution of the cation and anion in the nanofluidic channel (*l* = 500 nm, *σ* = 0.08 C m^−2^) with various channel sizes. For the channel with a smaller size, the selectivity of anions is better (Figure 4c–e). When *d* is 25 nm, the perm‐selectivity of nanofluidic channels almost disappears since the concentration curves of anions and cations overlap. The reason behind this phenomenon is that the range of overlapped electrical double layer (EDL) is large for narrow channels. The range is defined as Debye length:

(1) $$\lambda_{D}=\sqrt{\frac{\varepsilon \varepsilon_{0}RT}{2n_{\mathrm{bulk}}z^{2}F^{2}}}$$

where *ε* is the permittivity of water *ε*_0_ is the permittivity of a vacuum, *R* is the universal gas constant, *n*_bulk_ is the concentration of electrolyte solution, *T* is the absolute temperature, *z* is the valence number, and *F* is the Faraday's number.^[^ ^7^, ^38^ ^]^ When the range of EDL covers the physical dimensions of the channel, good selectivity is achieved.^[^ ^1^, ^12^ ^]^ High surface charge density also favors the improvement of ion selectivity (Figure S10a, Supporting Information). The output performance of channels with small channel size and high surface charge density is outstanding correspondingly (Figure S10b, Supporting Information). However, long channel length seems to weaken the energy conversion capability especially for narrow channels. As shown in Figure 4f, the output power of narrow channels (*d* = 5 nm) deteriorates with the increase of channel length. In theory, the maximal power is calculated as:

(2) $$P_{\max}=1/4G_{\mathrm{osm}}\varepsilon_{\mathrm{osm}}^{2}$$

where *G*_osm_ and *ε*_osm_ are the osmotic conductance and the osmotic potential, respectively. For LDH@AAO composite membranes, the bulk conductance dominates in osmotic conductance:^[^ ^1^ ^]^

(3) $$G_{\mathrm{osm}}\approx G_{\mathrm{bulk}}=\frac{\kappa \pi d^{2}}{4l}$$

where *G*_bulk_ and *κ* are the bulk conductance and the bulk conductivity, respectively. So a long channel length will limit the output power because of the low osmotic conductance. Besides, when the channel size is smaller, the decline of *G*_osm_ is larger with the increase of the channel length, which is the reason for the distinct deterioration of channels (*d* = 5 nm). It is worthy to mention that the output increases when the channel length is 550 nm for another two pore sizes. This may be attributed from the trade‐off between ion selectivity and osmotic conductance. As a result, the growth of LDH is a two‐edged sword for osmotic energy conversion and needs to be controlled at an appropriate degree.

### 2.5 The Effect of Environmental Factors

**Figure 5:**
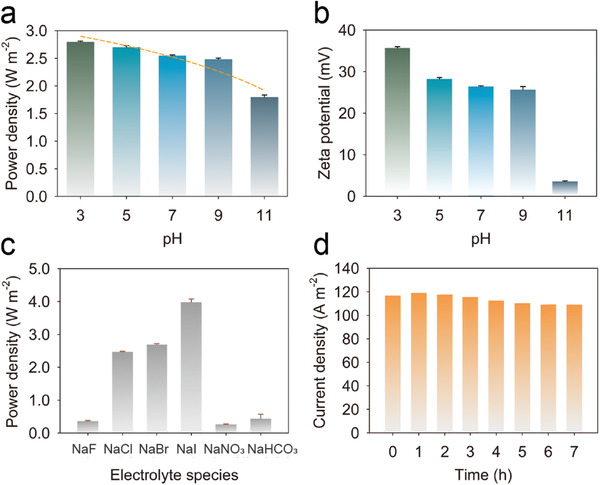
Effect of environmental factors on output performance of LDH@AAO composite membranes. a) Power generation in NaCl solutions at various pH values. Error bars represent SD (*n* = 3). b) Zeta potential of NiAl‐LDH at various pH values. Error bars represent SD (*n* = 3). c) Power generation under a series of sodium salts. Error bars represent SD (*n* = 3). d) The stability of LDH@AAO composite membranes under artificial seawater and river water without replenishing of electrolyte solution.

To explore the capability of composite membranes for practical application, we investigated the effect of environmental factors including the pH of electrolyte solutions, species of electrolyte, and working time. As the pH increases from 3 to 11, the power density decreases from 2.83 to 1.79 W m^−2^ (**Figure** **5**a). The variation results from the influence of pH value on the space charge and surface charge created by NiAl‐LDH nanoplates and AAO nanochannels.^[^ ^7^ ^]^ The measured zeta potential of NiAl‐LDH increased when the pH decreased (Figure 5b). The value of pH also exerts great influence on the amphoteric ‐OH groups on AAO channels. Thus, the positive surface charge density of these anion‐selective composite membranes is larger in acidic environment, contributing to better performance. We also compared the output power density of the composite membranes in various sodium salts to examine the selectivity of various anions. As shown in Figure 5c, the composite membranes exhibit the best output performance in NaI solutions (4.14 W m^−2^). We attribute this superiority to the large diffusion coefficient of I^−^ (2.045 × 10^−9^ m^2^ s^−1^). Since the composite membranes are anion‐selective, the fast diffusion of anion contributes to the efficient charge separation.^[^ ^39^ ^]^ Figure 5d recorded the change of the current density measured on the external circuit in a week without electrolyte replenishing. The slight attenuation (6.4%) demonstrates the stability of composite membranes in osmotic energy conversion. The ionic bonding between AAO and NiAl‐LDH contributes to the durability of the composite membranes.

### 2.6 Application of the Composite Membrane in Real‐Time Detection of Ion Concentration in Nutrient Solutions for Hydroponic Production

**Figure 6:**
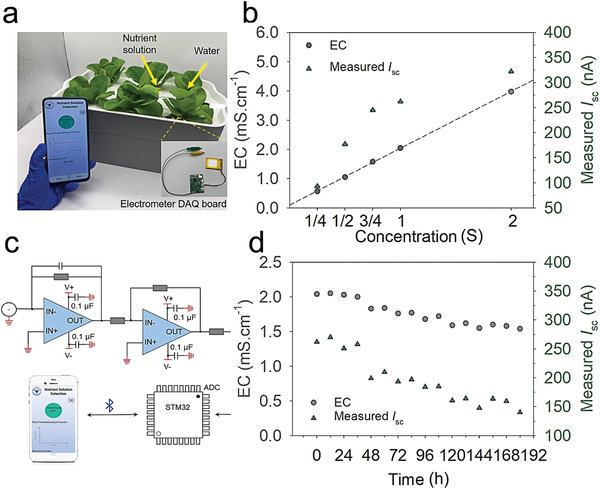
Application of the composite membrane in real‐time detection of ion concentration of nutrient solutions for hydroponic production. a) Photograph of the real‐time nutrient solution detecting system consisting of the LDH@AAO composite membrane and an electrometer DAQ board. The electrometer DAQ board acquires and processes the current signals from the composite membrane. Then the signals can be sent to APP on the phone via Bluetooth. b) The measured current and EC corresponding to the concentration of nutrient solutions. Error bars represent SD (*n* = 3). c) Schematic diagram of the electrometer DAQ board showing the information interaction process. d) Variation of *I*_sc_ measured from the real‐time nutrient solution detecting system during the growth of lettuce in a week. EC was also recorded as a reference for the actual concentration levels.

Considering the osmotic energy arises from variation in ion concentration, we explored the application of the LDH@AAO composite membrane in detecting ion concentration of nutrient solutions. A real‐time nutrient solution detecting system based on LDH@AAO composite membranes was designed (**Figure** **6**a). The composite membrane was fixed on the side of a cuboid box using epoxy (Figure S11, Supporting Information). The box acted as containers for nutrient solutions and was surrounded by water (Figures S12 and S13b, Supporting Information). The salinity gradient energy between nutrient solutions and water was converted into electricity. Two Ag/AgCl electrodes were distributed on two sides of the box and connected to an electrometer data acquisition (DAQ) board which could acquire and process the signals from nanofluidic channels (Figure S13a, Supporting Information). Figure 6c demonstrates the information interaction process. The electrometer DAQ board sends the current signal to a customized application (APP) on the smartphone via Bluetooth. The composite membrane was fixed between nutrient solutions with different concentrations and ionized water. Hoagland's nutrient solution is used in nutrient detection, which has been widely applied in hydroponic production. The concentration of standard nutrient solution is set as 1 strength (S). Figure 6b recorded the measured short‐circuit current (*I*_sc_) and EC under various concentrations. EC is proportional to the concentration while the measured current positively correlates with the concentration. So the *I*_sc_ can act as a measurement of the ion concentration level and the real‐time detection can be realized through recording real‐time *I*_sc_ generated from the composite membrane. For instance, we chose 0.27 and 0.10 µA as the upper and lower limits of measured current in the following test, which are in accordance with 1 S and 1/4 S, respectively. Figure S14, Supporting Information, shows current curves and corresponding warnings for four concentrations in 60 s. The APP can estimate the concentration level according to the appropriate range which was set before and warn users to adjust the concentration of nutrient solutions in time when the level is low or high (Video S1, Supporting Information). For real application, a long‐term monitoring of actual hydroponic production was carried out. Seedlings of romaine lettuce were hydroponically cultivated during a week (Figure S15, Supporting Information). The *I*_sc_ was measured per 12 h during 7 d and the cuboid box was immersed in the tank all the time. To ensure the overall volume of nutrient solution to be the same during the test, a certain volume of water was added to the nutrient solution daily. EC was also measured and used as a reference for the real concentration level of nutrient solution. Figure 6d exhibits the variation of the measured *I*_sc_ and EC with the growth of lettuce, comparing the difference between measured nutrient concentration and the actual concentration. It is evident that the variation of measured *I*_sc_ can reflect the level and changing trend of nutrient solution concentration. However, after immersion in nutrient solutions for 7 d, the composite membrane was partly fouled on the outer surface (Figure S16, Supporting Information). It turns out that the output performance of composite membrane undergoes a deterioration (12.8%) after comparing *I*_sc_ in standard nutrient solution (1 S) with the value obtained at 0 d. The antifouling property of LDH@AAO composite membrane remains to be improved for future application.

## 3 Conclusion

In conclusion, we adopted a precipitant‐free and simple in situ growth method without chemical modification to fabricate LDH@AAO composite membranes for osmotic energy harvesting. Positive surface charges of LDH layers endow the composite membrane with excellent selectivity of anions. Both experiments and theoretical simulations certify the vital role of LDH growth in ion distribution and osmotic energy conversion. The fabricated membrane can achieve a power density of 2.85 W m^−2^ by mixing artificial seawater and river water. The output performance is distinctive among anion‐selective nanofluidic membranes. Intriguingly, we designed a real‐time detecting system based on LDH@AAO composite membranes for the nutrient solution. Current signals generated from variation in ion concentration of nutrient solutions were employed to measure concentration levels and warn users to adjust nutrient solutions in time. Our work provides insights into naturally anion‐selective membranes for osmotic energy harvesting and explores novel applications of nanofluidic channels in hydroponic production. It can be envisioned that more precise detection of specific ion concentration is one of the promising directions for the application of osmotic energy harvesters.

## 4 Experimental Section

### Chemicals

The AAO membranes were purchased from Hefei Pu‐Yuan Nano Technology, Ltd., China. Hoagland's nutrient solution was purchased from Shenzhen Konong Horticulture Technology, Ltd., China. Other chemicals were all analytical grade and obtained from Sinopharm Chemical Reagent Co. Ltd., China. All solutions were prepared using Milli‐Q water (18.2 MΩ cm).

### Fabrication of LDH@AAO Composite Membranes

The composite membrane was fabricated based on an in situ growth technique. AAO plates were vertically fixed in beakers containing Ni(NO_3_)_2_ 6H_2_O aqueous solutions (1 m). These beakers were then heated in water bath. The temperature was controlled at 80 °C. The pH of Ni(NO_3_)_2_ 6H_2_O aqueous solutions was adjusted by nitric acid or ammonia solution. The obtained composite membrane was dried at room temperature for 24 h after the rinse with deionized water.

### Characterization

SEM images were captured using SEM (Gemini 300, ZEISS, Oberkochen, Germany). The XPS was performed in VG ESCALAB MARK II. XRD was performed in a diffractometer (D8 Advance, Bruker, Karlsruhe, Germany). Nitrogen sorption isotherms were obtained using TriStar II Plus (Micromeritics, Norcross, GA, USA). The pore distribution was acquired from the desorption branch based on the BJH model. Contact angles were measured in Theta Lite (Biolin, Stockholm, Sweden). To obtain the element distribution on the cross‐section of LDH@AAO composite membrane, EDX spectroscopy was conducted. Zeta potential of NiAl‐LDH was analyzed in a solution system using Zetasizer Nano ZS90 (Malvern, Worcestershire, UK).

### Electrical Measurements

The composite membrane was fixed between a customized electrochemical cell. The ion transport properties and energy conversion capabilities were acquired with a Keithley 6487 semiconductor picoammeter (Keithley, Cleveland, OH, USA). A pair of Ag/AgCl electrodes were employed to apply the transmembrane electrical potential and measure the resistance and current. During the detection of *I*‐*V* curves under various concentration gradients, the range of the sweeping voltage was −0.2 to 0.2 V, with a step voltage of 0.02 V. A pair of agar‐saturated potassium chloride salt bridge was used to eliminate the imbalanced electrode potential. The effective working area of the membrane was set as 3 × 10^4^ µm^2^. The conductance value of bulk NaCl solution was obtained by measuring the conductance of a neutral charged silicon membrane in the customized electrochemical cell. The pH of electrolyte solutions was adjusted by HCl and NaOH solutions. During the stability test, the composite membrane was fixed in the electrochemical cell and the electrolyte solutions were not refreshed.

### Statistical Analysis

No filtering was applied in the experimental results presented in this work. For measuring conductance versus salt concentration, experimental results presented used one composite membrane (sample size *n* = 1). For measuring current‐voltage curve, experimental results presented used *n* = 1. For measuring *E*_diff_ and *I*_diff_ under various concentration gradients, three samples were tested (*n* = 3). For measuring the current density as the function of load resistance, one composite membrane was used (*n* = 1). For measuring the power density corresponding to different growth pH and reaction times, the data were presented as the mean ±SD of three independent experiments (*n* = 3). For measuring power density under various environmental conditions, three samples were tested (*n* = 3). For measuring the stability of the composite membrane, one sample was used (*n* = 1). For nutrient solution detection, one cuboid box with LDH@AAO composite membrane was employed (n = 1).

## Conflict of Interest

The authors declare no conflict of interest.

## Supporting information

Supporting Information

Supporting Video 1

## Data Availability

The data that support the findings of this study are available from the corresponding author upon reasonable request.

## References

[1] M. Macha , S. Marion , V. V. R. Nandigana , A. Radenovic , Nat. Rev. Mater. 2019, 4, 588.

[2] D. Pakulski , W. Czepa , S. Del Buffa , A. Ciesielski , P. Samor , Adv. Funct. Mater. 2020, 30, 1902394.

[3] Z. Zhang , L. Wen , L. Jiang , Chem. Soc. Rev. 2018, 47, 322. 29300401 10.1039/c7cs00688h

[4] J. Gao , X. Liu , Y. Jiang , L. Ding , L. Jiang , W. Guo , Small 2019, 15, 1804279. 10.1002/smll.201804279 30653272

[5] Z. Zhang , L. Wen , L. Jiang , Nat. Rev. Mater. 2021, 6, 622.

[6] C. Zhu , P. Liu , B. Niu , Y. Liu , W. Xin , W. Chen , X. Y. Kong , Z. Zhang , L. Jiang , L. Wen , J. Am. Chem. Soc. 2021, 143, 1932. 33455164 10.1021/jacs.0c11251

[7] W. Xin , Z. Zhang , X. Huang , Y. Hu , T. Zhou , C. Zhu , X.‐Y. Kong , L. Jiang , L. Wen , Nat. Commun. 2019, 10, 3876. 31462636 10.1038/s41467-019-11792-8 PMC6713777

[8] H. Wang , L. Su , M. Yagmurcukardes , J. Chen , Y. Jiang , Z. Li , A. Quan , F. M. Peeters , C. Wang , A. K. Geim , S. Hu , Nano Lett. 2020, 20, 8634. 33179495 10.1021/acs.nanolett.0c03342

[9] C. Chen , D. Liu , L. He , S. Qin , J. Wang , J. M. Razal , N. A. Kotov , W. Lei , Joule 2020, 4, 247.

[10] S. Hong , F. Ming , Y. Shi , R. Li , I. S. Kim , C. Y. Tang , H. N. Alshareef , P. Wang , ACS Nano 2019, 13, 8917. 31305989 10.1021/acsnano.9b02579

[11] W. Guo , C. Cheng , Y. Wu , Y. Jiang , J. Gao , D. Li , L. Jiang , Adv. Mater. 2013, 25, 6064. 23900945 10.1002/adma.201302441

[12] L. Ding , D. Xiao , Z. Lu , J. Deng , Y. Wei , J. Caro , H. Wang , Angew. Chem., Int. Ed. 2020, 59, 8270. 10.1002/anie.201915993 31950586

[13] M. Caglar , I. Silkina , B. T. Brown , A. L. Thorneywork , O. J. Burton , V. Babenko , S. M. Gilbert , A. Zettl , S. Hofmann , U. F. Keyser , ACS Nano 2020, 14, 2729. 31891480 10.1021/acsnano.9b08168 PMC7098055

[14] H. Gao , W. Chen , C. Xu , S. Liu , X. Tong , Y. Chen , Environ. Sci. Technol. 2020, 54, 2931. 32048835 10.1021/acs.est.9b05100

[15] Y. Fu , X. Guo , Y. Wang , X. Wang , J. Xue , Nano Energy 2019, 57, 783.

[16] Z. Wu , P. Ji , B. Wang , N. Sheng , M. Zhang , S. Chen , H. Wang , Nano Energy 2021, 80, 105554.

[17] Q. Y. Wu , C. W. Wang , R. L. Wang , C. J. Chen , J. L. Gao , J. Q. Dai , D. P. Liu , Z. W. Lin , L. B. Hu , Adv. Energy Mater. 2020, 10, 1902590.

[18] G. M. Tomboc , J. Kim , Y. T. Wang , Y. Son , J. H. Li , J. Y. Kim , K. Lee , J. Mater. Chem. A 2021, 9, 4528.

[19] J. C. Munyemana , J. Chen , Y. X. Han , S. S. Zhang , H. D. Qiu , Microchim. Acta 2021, 188, 80. 10.1007/s00604-021-04739-8 33576899

[20] W. Yu , N. P. Deng , K. W. Cheng , J. Yan , B. W. Cheng , W. M. Kang , J. Energy Chem. 2021, 58, 472.

[21] J. Yu , F. Yu , M. F. Yuen , C. D. Wang , J. Mater. Chem. A 2021, 9, 9389.

[22] J. X. Li , B. Li , J. K. Wang , L. He , Y. F. Zhao , Acta Chim. Sin. 2021, 79, 238.

[23] K. J. Walters , B. K. Behe , C. J. Currey , R. G. Lopez , Hortscience 2020, 55, 758.

[24] U. Samarakoon , P. A. W. , W. A. P. Weerakkody , Trop. Agric. Res. 2006, 18, 13.

[25] A. P. Montoya , F. A. Obando , J. A. Osorio , J. G. Morales , M. Kacira , Comput. Electron. Agric. 2020, 178, 105758.

[26] L. Xue , Z. Lu , Y. Cheng , X. Sun , H. Lin , X. Xiao , X. Liu , S. Zhuo , Chem. Commun. 2018, 54, 8494. 10.1039/c8cc04162h 30003201

[27] K. H. Goh , T. T. Lim , Z. Dong , Water Res. 2008, 42, 1343. 18061644 10.1016/j.watres.2007.10.043

[28] H. Zeng , Z. Yu , Y. Peng , L. Zhu , Appl. Clay Sci. 2019, 183, 105322.

[29] Y. Zhao , J. Wang , X.‐Y. Kong , W. Xin , T. Zhou , Y. Qian , L. Yang , J. Pang , L. Jiang , L. Wen , Natl. Sci. Rev. 2020, 7, 1349. 34692163 10.1093/nsr/nwaa057 PMC8288931

[30] L. Ding , D. Xiao , Z. Lu , J. Deng , Y. Wei , J. Caro , H. Wang , Angew. Chem., Int. Ed. 2020, 59, 8720. 10.1002/anie.201915993 31950586

[31] Z. Zhang , L. He , C. Zhu , Y. Qian , L. Wen , L. Jiang , Nat. Commun. 2020, 11, 875. 32054863 10.1038/s41467-020-14674-6 PMC7018769

[32] X. Zhu , J. Zhong , J. Hao , Y. Wang , J. Zhou , J. Liao , Y. Dong , J. Pang , H. Zhang , Z. Wang , W. Zhang , W. Zheng , Z. Jiang , Y. Zhou , L. Jiang , Adv. Energy Mater. 2020, 10, 2001552.

[33] W. Xin , H. Xiao , X. Y. Kong , J. Chen , L. Yang , B. Niu , Y. Qian , Y. Teng , L. Jiang , L. Wen , ACS Nano 2020, 14, 9701. 32687698 10.1021/acsnano.0c01309

[34] Z. Zhang , S. Yang , P. Zhang , J. Zhang , G. Chen , X. Feng , Nat. Commun. 2019, 10, 2920. 31266937 10.1038/s41467-019-10885-8 PMC6606750

[35] X. Zhu , J. Hao , B. Bao , Y. Zhou , H. Zhang , J. Pang , Z. Jiang , L. Jiang , Sci. Adv. 2018, 4, eaau1665. 30397649 10.1126/sciadv.aau1665 PMC6203222

[36] J. Gao , W. Guo , D. Feng , H. Wang , D. Zhao , L. Jiang , J. Am. Chem. Soc. 2014, 136, 12265. 25137214 10.1021/ja503692z

[37] Z. Zhang , X. Sui , P. Li , G. Xie , X. Y. Kong , K. Xiao , L. Gao , L. Wen , L. Jiang , J. Am. Chem. Soc. 2017, 139, 8905. 28602079 10.1021/jacs.7b02794

[38] C. G. Gray , P. J. Stiles , Eur. J. Phys. 2018, 39, 053002.

[39] H. C. Zhang , J. Hou , Y. X. Hu , P. Y. Wang , R. W. Ou , L. Jiang , J. Z. Liu , B. D. Freeman , A. J. Hill , H. T. Wang , Sci. Adv. 2018, 4, eaaq0066. 29487910 10.1126/sciadv.aaq0066 PMC5817922

[40] J. Q. Kang , Y. Zhou , Y. Feng , X. Chen , J. Yuan , W. Guo , Y. Wei , L. Jiang , Adv. Funct. Mater. 2017, 27, 1603623.

