# Supplementary material for: Anion‐Selective Layered Double Hydroxide Composites‐Based Osmotic Energy Conversion for Real‐Time Nutrient Solution Detection
Source: Adv Sci (Weinh). 2022 Jan 6;9(6):2103696. doi: 10.1002/advs.202103696 (PMC8867156; doi:10.1002/advs.202103696)
Supplement: Supplementary file 1 — Supporting Information [file ADVS-9-2103696-s002.pdf]

ADVANCED SCIENCE
Open Access

Supporting Information

for *Adv. Sci.*, DOI: 10.1002/advs.202103696

Anion-selective layered double hydroxide composites-based osmotic energy conversion for real-time nutrient solution detection

*Yaqian Liu, Jianfeng Ping, and Yibin Ying**

Supporting Information

Anion-selective layered double hydroxide composites-based osmotic energy conversion for real-time nutrient solution detection

Yaqian Liu, Jianfeng Ping, Yibin Ying*

Laboratory of Agricultural Information Intelligent Sensing, School of Biosystems Engineering and Food Science, Zhejiang University, Hangzhou, Zhejiang 310058, China

Corresponding author: Prof. Yibin Ying

E-mail: ybying@zju.edu.cn

Supporting Note 1 Energy Conversion Efficiency

For a given concentration gradient, the anion transference number (*i.e.*, t_-) can be calculated as[1]:

$$t_-=\frac{1}{2}\left(\frac{E_{diff}}{\frac{RT}{zF}\ln\left(\frac{\gamma_{cH}c_H}{\gamma_{cL}c_L}\right)}+1\right) \quad (1)$$

Here, E_{diff}, R, T, z, F, γ, and c represent the diffusion potential, universal gas constant, absolute temperature, charge number, Faraday constant, activity coefficient of ions, and ion concentration, respectively. The energy conversion efficiency is defined as the ratio of the output energy (electrical energy) to the input energy (Gibbs free energy of mixing). The energy conversion efficiency of the maximum power generation can be calculated as[2]:

$$\eta_{max}=\frac{1}{2}(2t_--1)^2 \quad (2)$$

Supporting Note 2 Numerical simulation

The ion concentration distribution and osmotic power generation under the growth of LDH on AAO is investigated using a commercial finite-element software package COMSOL Multiphysics. Through setting appropriate boundary parameters, the numerical simulation based on coupled Poisson and Nernst-Planck equations was carried out. According to Nernst-Planck equation, the ionic flux (J_i) is calculated as:

$$\boldsymbol{J}_i=D_i\nabla c_i+\frac{z_iF}{RT}D_ic_i\nabla\varphi \quad (5)$$

where $\boldsymbol{J}_i$, D_i, c_i, φ, z_i, R, F, and T are the ionic flux, diffusion coefficient, ion concentration, electrical potential, the valence, universal gas constant, Faraday constant, and absolute temperature, respectively.

The system is simplified in a steady-state condition, and the ion flux satisfies the time-independent continuity:

$$\nabla\cdot\boldsymbol{J}_i=0 \quad (6)$$

The Poisson equation gives the relationship between the electric potential and ion concentration:

$$\nabla^2\varphi=-\frac{F}{\varepsilon}\sum_i z_ic_i \quad (7)$$

where ε is permittivity of the fluid. The coupled equations (5) to (7) could be solved with finite-element calculations for the ion concentration distribution with the given geometry and

appropriate boundary conditions.

As shown in Figure S9, the fluidic pathway of the LDH@AAO composite membrane is simplified to be a single 2D channel. To minimize the influence of the resistance of mass transfer at the entrance and exit, two electrolyte reservoirs (400×200 nm) are added. Considering the ion flux has the zero normal components at boundaries:

$$\mathbf{n}\cdot\boldsymbol{J}_{\mathrm{i}}=0 \quad (8)$$

so the ionic current through the nanochannel can be calculated:

$$\mathrm{I}=\int_{\mathrm{s}} F(z_{\mathrm{p}}\boldsymbol{J}_{\mathrm{p}}+z_{\mathrm{n}}\boldsymbol{J}_{\mathrm{n}})\cdot\mathbf{n}\mathrm{d}S \quad (9)$$

The external potential from -0.2 V to 0.2 V is applied across the channel, and the corresponding power output can be obtained from the calculated current-voltage curves. KCl solution is used in this simulation, and the concentration gradient is set to be 50-fold (*i.e.* 0.5 M /0.01 M). The channel size (d), the channel length (l), and surface charge density (σ) are set to be within reasonable range.

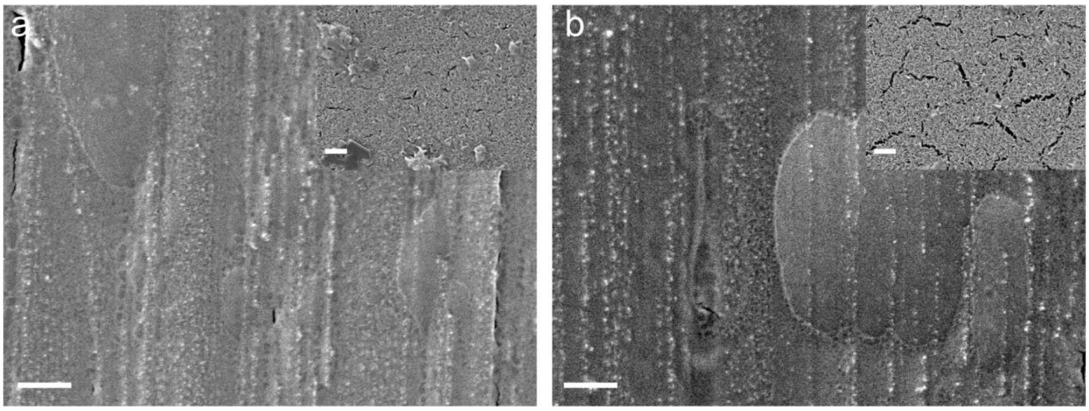

Figure S1. SEM images of LDH@AAO composite membranes employing AAO templates with different channel sizes, including (a) 80-100 nm and (b) 110-150 nm (scale bar: 1 μm). When the reaction begins, the nucleation and growth rates of LDH are quicker on the outer surface than on the inner surface because the outer surface of AAO is exposed more sufficiently to the reaction solution. An increasing number of Ni^{2+} ions are prevented from diffusing into the inner surfaces because of the decreased size of the pores. The relatively high concentration of Ni^{2+} ions on the outer surface accelerate the adsorption of Ni^{2+} ions and formation of LDH plates. In contrast, the low concentration of Ni^{2+} ions on the inner surface is unfavorable for the growth of LDH but makes it easy for the formation of boehmite.

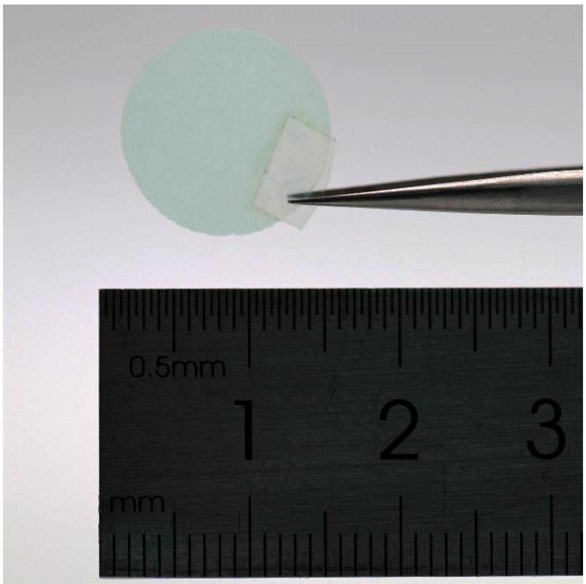

Figure S2. Photograph of the LDH@AAO composite membrane.

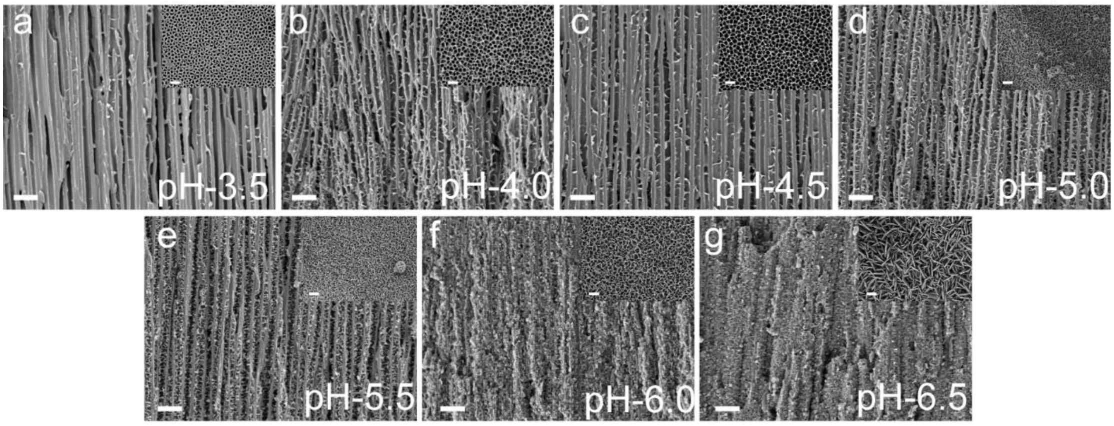

Figure S3. SEM images of composite membranes fabricated in reaction solutions with different pH values, including (a) pH=3.5, (b) pH=4.0, (c) pH=4.5, (d) pH=5.0, (e) pH=5.5, (f) pH=6.0, and (g) pH=6.5 (scale bar: 1 μm). With the increase of the pH, the reaction solution is more favorable to the adsorption of Ni^{2+} and the NiAl-plates becomes larger and thicker on the surface of AAO.[5] When pH ≤ 5.5, the unblocked pores of AAO permit the diffusion of Ni^{2+} into the nanochannels while when pH ≥ 6.0, the dense distribution of LDH plates on the surface of AAO hinders the diffusion of Ni^{2+} into nanochannels and prevents the growth of LDH plates along nanochannels.

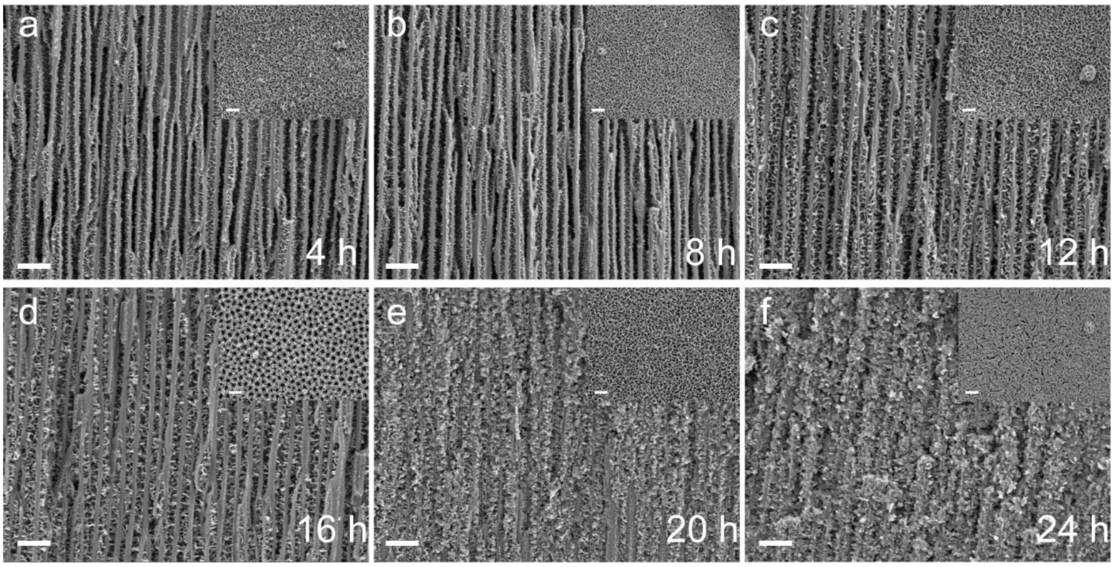

Figure S4. SEM images of composite membranes fabricated with different reaction time, including (a) 4 h, (b) 8 h, (c) 12 h, (d) 16 h, (e) 20 h, and (f) 24 h (scale bar: 1 μm). When the reaction begins, the nucleation and growth rates of LDH are quicker on the outer surface than on the inner surface since the outer surface of AAO is exposed more sufficiently to the reaction solution.[6] After plenty of LDH plates are formed on the outer surface, the diffusion of Ni^{2+} into the nanochannels is limited, which is the reason for the difference in cross-sectional SEM images after 20 h.

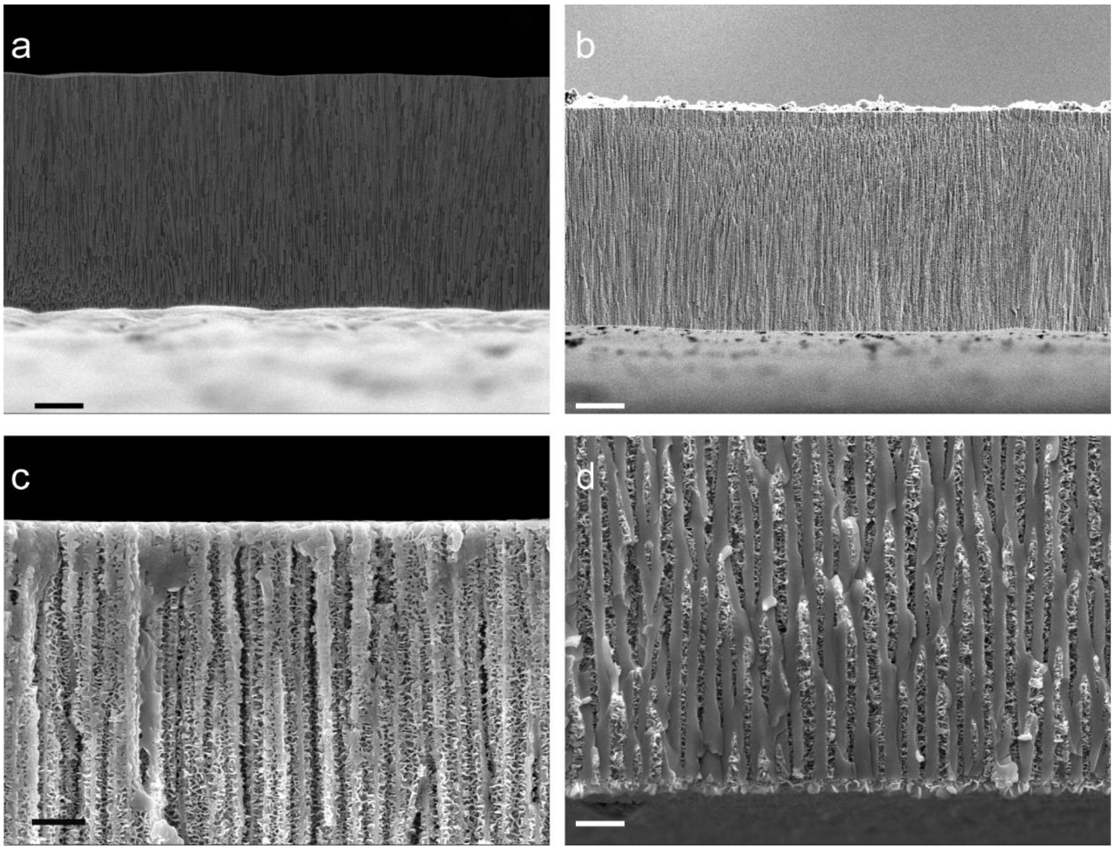

Figure S5. (a) Cross-sectional SEM image of the AAO template (scale bar: 10 μm). (b) Cross-sectional SEM image of the LDH@AAO composite membrane (scale bar: 10 μm). (c) Cross-sectional SEM image of the top of the LDH@AAO composite membrane (scale bar: 1 μm). The average thickness of LDH layers is 457 nm. (d) Cross-sectional SEM image of the bottom of the LDH@AAO composite membrane (scale bar: 1 μm). The average thickness of LDH layers is 406 nm.

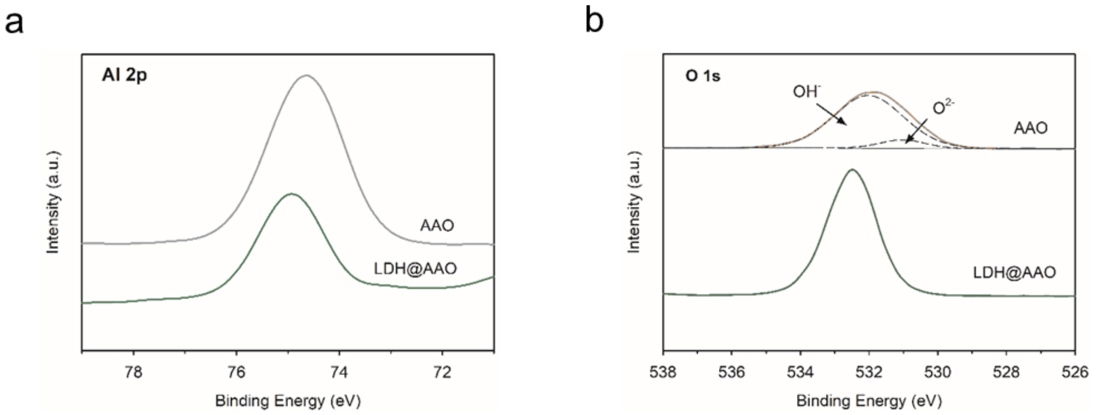

Figure S6. The XPS results of the AAO template and LDH@AAO composite membrane including Al 2p (a) and O 1s (b).

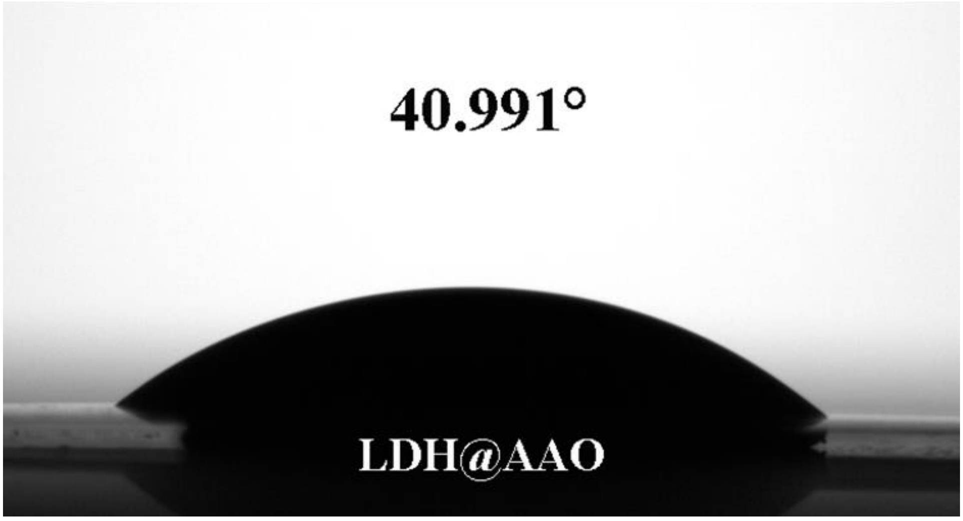

Figure S7. Contact angle measurement of the LDH@AAO composite membrane.

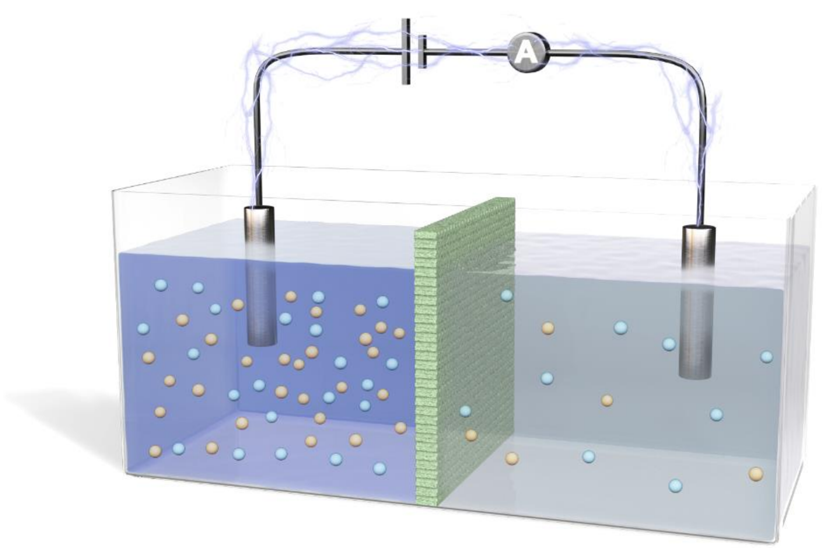

Figure S8. Schematic of the electrochemical cell for evaluation of surface-charge-governed ion transport property.

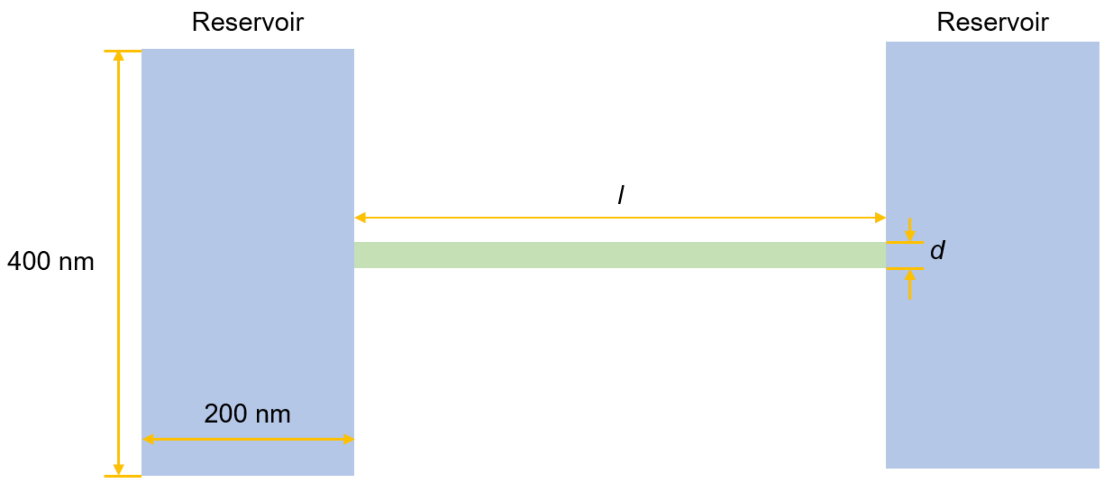

Figure S9. Model of theoretical simulation (Drawing not to scale).

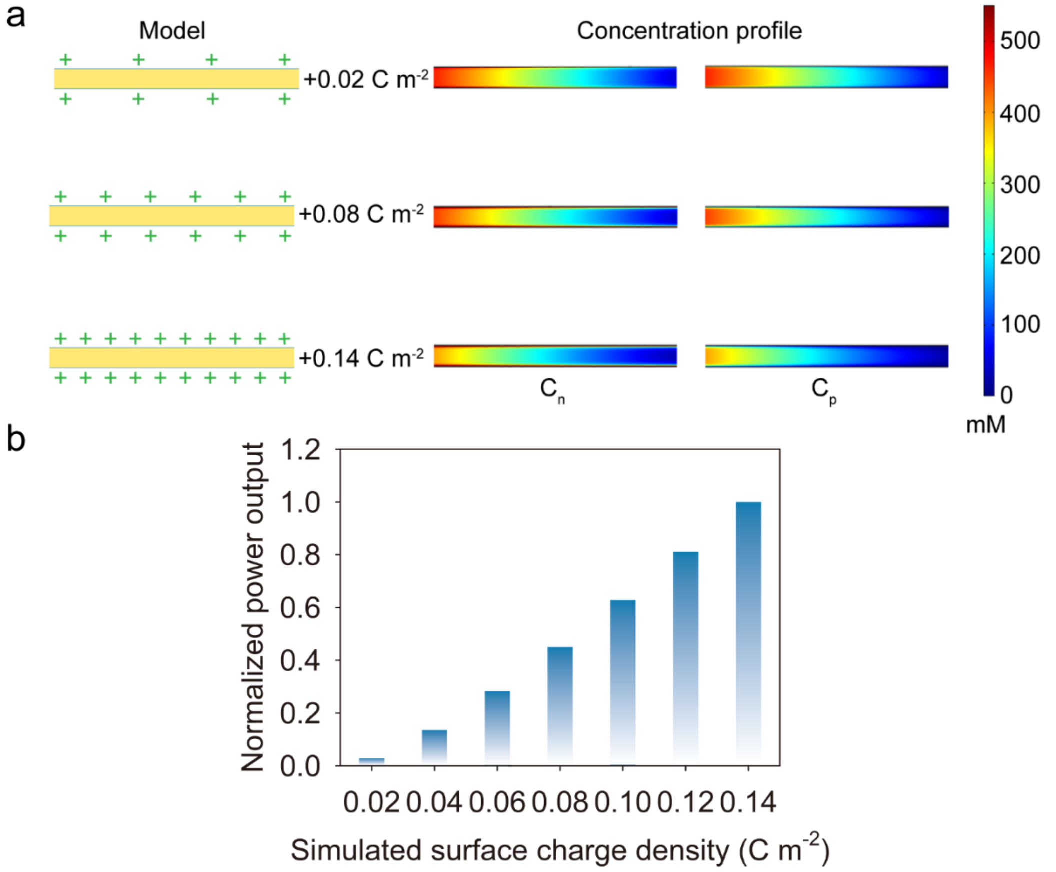

Figure S10. (a) The ionic concentration profiles of nanofluidic channels (*l* =500 nm, *d*=15 nm) with various surface charge densities based on numerical simulations. (b) The calculated mormalized power output of nanofluidic channels (*l* =500 nm, *d*=5 nm) with various surface charge densities.

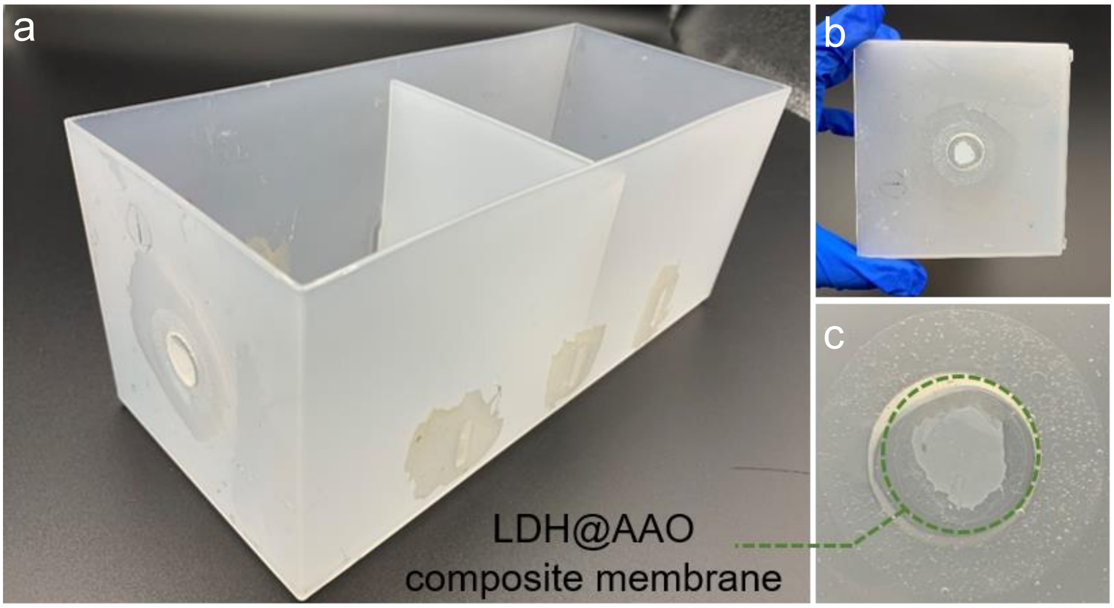

Figure S11. The photograph of the cuboid with the composite membrane fixed on the side.

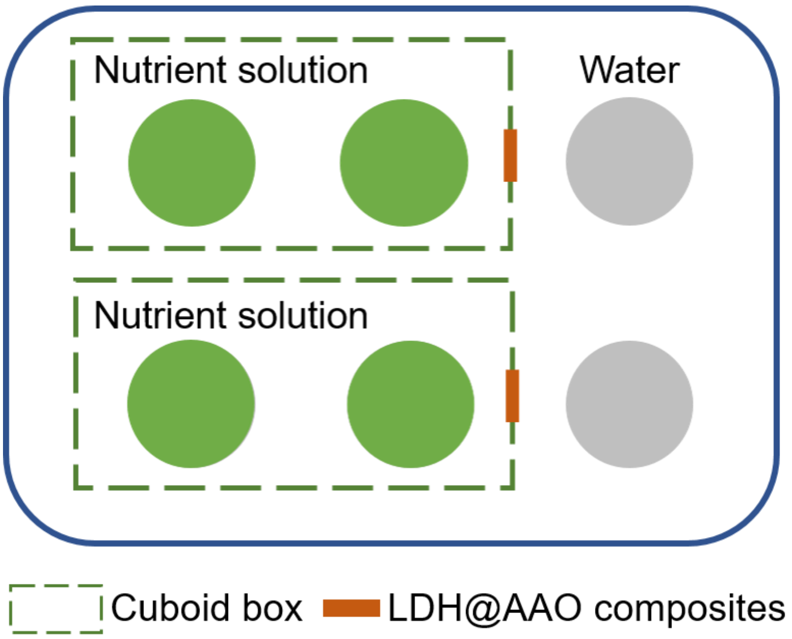

Figure S12. Schematic showing the inner structure of the hydroponic production system.

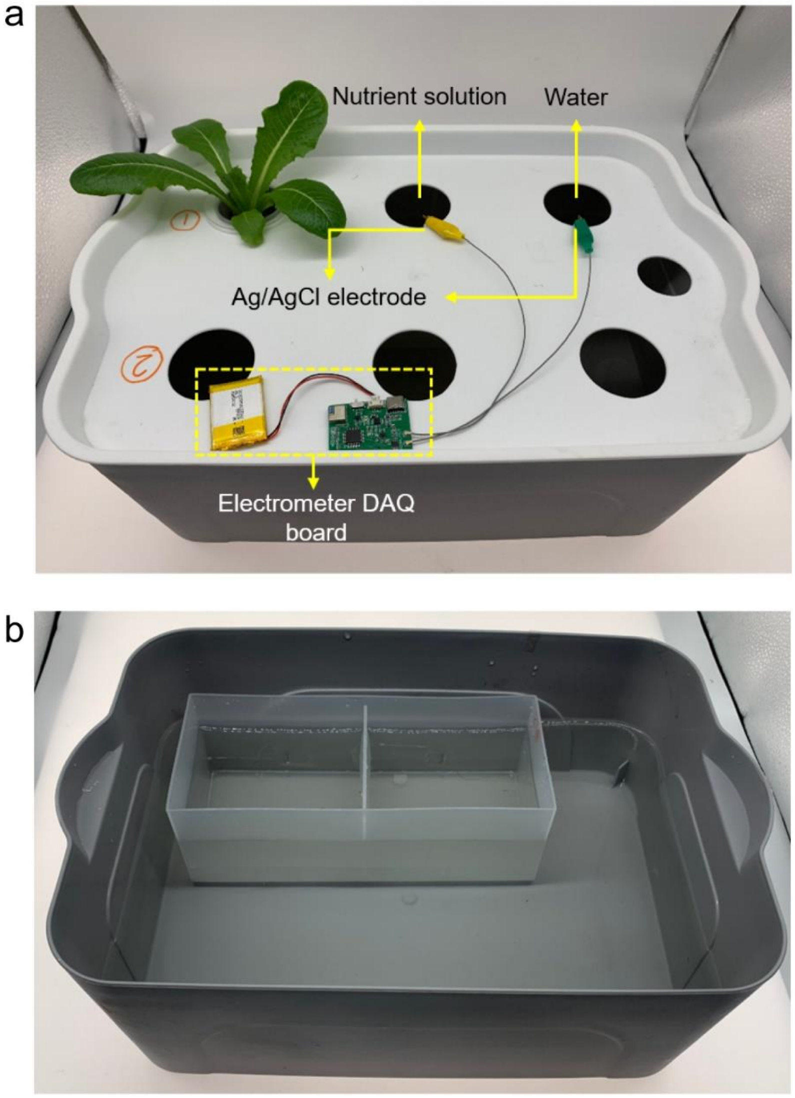

Figure S13. Photographs showing the actual structure of the hydroponic production system. (a) The electrometer DAQ board is connected to two Ag/AgCl electrodes which are immersed into nutrient solution and water separately. Plants can be cultivated in pores shown in the picture. (b) The inner structure of the hydroponic production system, where the tank is filled with Milli-Q water and the cuboid box is filled with nutrient solution. The number of cuboid box can be 2 in real application.

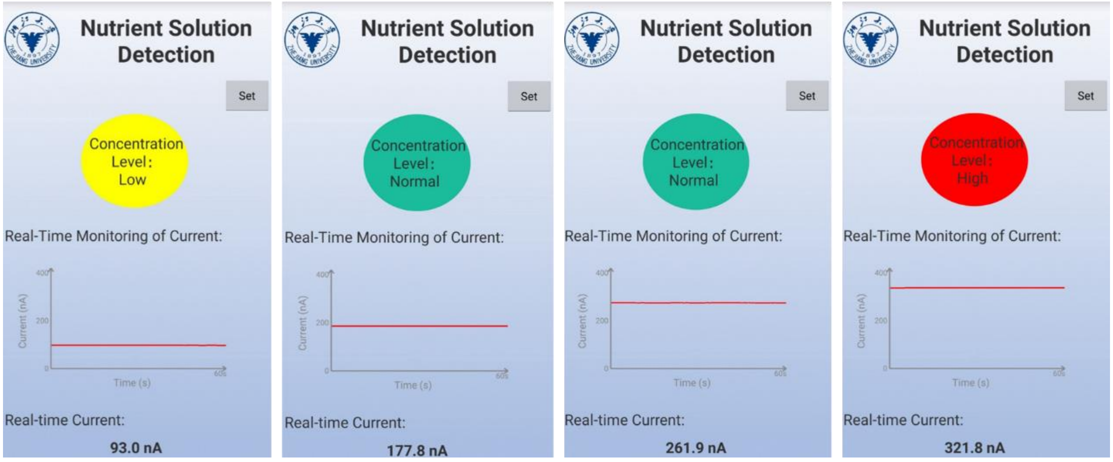

Figure S14. Recorded plot (time versus current) screens under different concentration levels of nutrient solution. When the measured current is higher (lower) than the preset threshold, the APP will warn users to adjust the concentration of nutrient solution in red (yellow) color.

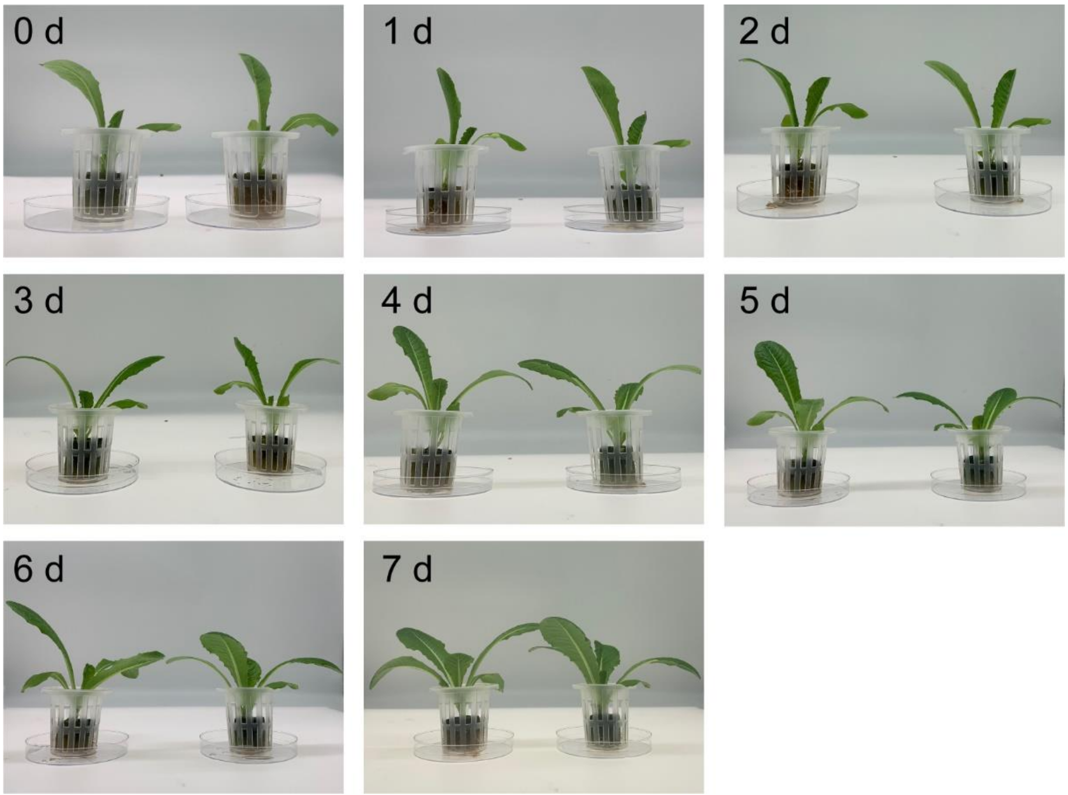

Figure S15. Photographs of growth of romaine lettuces, which were hydroponically cultivated during a week.

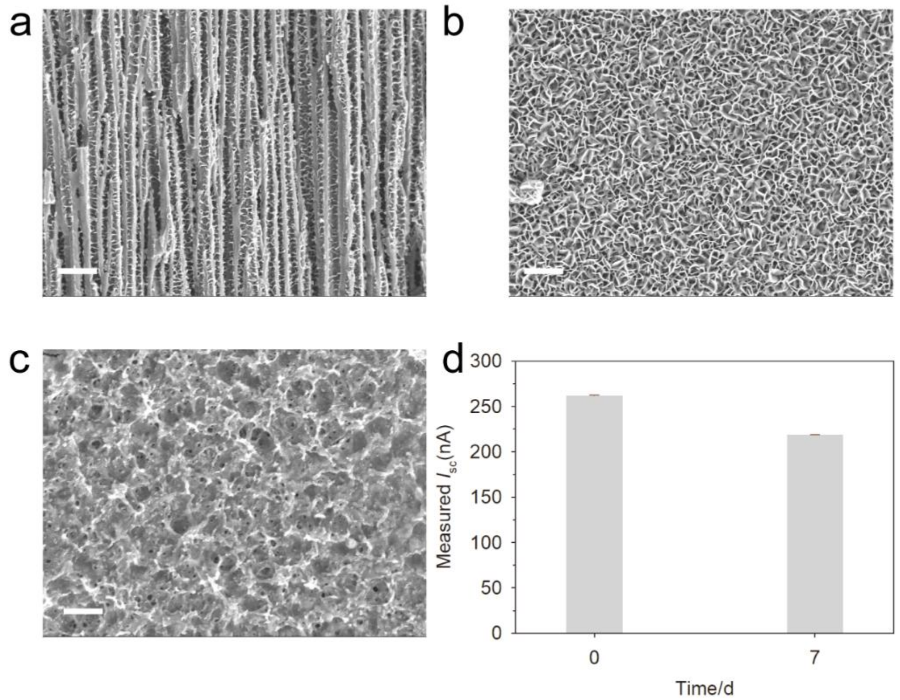

Figure S16. Characterization of LDH@AAO composite membrane after detecting concentration of nutrient solution for 7 d. (a) SEM image of cross-sectional part of composite membrane (scale bar: 1 μm). (b) SEM image of outer surface of composite membrane contacted with water (scale bar: 1 μm). (c) SEM image of outer surface of composite membrane contacted with nutrient solution (scale bar: 1 μm). (d) Comparison of measured *I*sc in standard nutrient solution (1S) at 0 d and 7 d.

References

[1] W. Xin, H. Xiao, X. Y. Kong, J. Chen, L. Yang, B. Niu, Y. Qian, Y. Teng, L. Jiang, L. Wen, *ACS Nano* **2020**, 14, 9701.

[2] D.-K. Kim, C. Duan, Y.-F. Chen, A. Majumdar, *Microfluid. Nanofluid.* **2010**, 9, 1215.
